# Intrauterine Infection With Coxsackievirus: Is it a Cause of Congenital Cardiac Malformations?

**DOI:** 10.1155/S1064744995000366

**Published:** 1995

**Authors:** William J. Watson, Sami Awadallah, Mary Jo Jaqua

**Affiliations:** 1 Department of Perinatal Medicine Sioux Valley Hospital University of South Dakota School of Medicine 1201 S. Euclid Avenue, Ste. 204 Sioux Falls SD 57105 USA; 2 Division of Pediatric Cardiology Department of Pediatrics University of South Dakota School of Medicine Sioux Falls SD USA; 3 Department of Laboratory Medicine University of South Dakota School of Medicine Sioux Falls SD USA

## Abstract

*Background:* Although maternal infections with coxsackievirus during pregnancy are relatively common, fetal infections are quite rare. Coxsackievirus infection in utero has been associated with myocarditis, but has not been proven a teratogen.

*Case:* A patient whose fetus had structural cardiac anomalies and hydrops was found to have an intrauterine infection with Coxsackie B-1 virus, proven by virus isolation from the amniotic fluid. This infection led to increasing intrauterine hydrops and subsequent neonatal death.

*Conclusion:* This interesting association of intrauterine infection with Coxsackie B virus and structural cardiac anomalies in the fetus warrants further investigation.


Infectious Diseases in Obstetrics and Gynecology 3:79–81 (1995)
© 1995 Wiley-Liss, Inc.

### Intrauterine Infection With Coxsackievirus: Is It a Cause of Congenital Cardiac Malformations?

**William J. Watson, Sami Awadallah, and Mary Jo Jaqua**

*Department of Perinatal Medicine, Sioux Valley Hospital (W.J.W.), and Division of Pediatric Cardiology, Department of Pediatrics (S.A.), and Department of Laboratory Medicine (M.J.J.), University of South Dakota School of Medicine, Sioux Falls, SD*

Abstract

***Background:*** **Although maternal infections with coxsackievirus during pregnancy are relatively common, fetal infections are quite rare. Coxsackievirus infection in utero has been associated with myocarditis, but has not been proven a teratogen.**

***Case:*** **A patient whose fetus had structural cardiac anomalies and hydrops was found to have an intrauterine infection with Coxsackie B-1 virus, proven by virus isolation from the amniotic fluid. This infection led to increasing intrauterine hydrops and subsequent neonatal death.**

***Conclusion:*** **This interesting association of intrauterine infection with Coxsackie B virus and structural cardiac anomalies in the fetus warrants further investigation.** © 1995 Wiley-Liss, Inc.

Key words

Coxsackie B-1 virus, cardiac disease—congenital, pregnancy, viral infection, enterovirus

Enterovirus infections during pregnancy are relatively common. The NIH Collaborative Perinatal Project found that serologic evidence of infection with group B coxsackievirus occurred in approximately 9% of 198 unselected women studied during pregnancy.[1] There is evidence that seroconversion may occur even more frequently in the peak enterovirus season.[2] Maternal enterovirus infections in pregnancy show a wide variety of symptomatology, ranging from no clinical illness, to an upper-respiratory-type infection to a febrile disease characterized by lower abdominal pain.

Most enterovirus infections occurring during pregnancy result in the delivery of unaffected, healthy infants. Experimental research has shown that the transplacental passage of a virus does not easily occur.[3] In 1986, Modlin[4] concluded that no more than 24 cases of viral infection in utero could be documented in the medical literature. However, several reports have indicated that enterovirus infection may occur by a transplacental route. Basso et al.[5] isolated both echovirus and coxsackievirus from placental and fetal tissues after maternal infection.

Rosenberg,[6] in a recent review on the cardiovascular effects of congenital infections, pointed out that rubella virus infection in utero has been established as a cause of fetal cardiovascular malformations, but that other viruses have not been clearly established as a cause of these malformations. We present in this report a case of proven intrauterine infection with Coxsackie B-1 virus in which the fetus was noted to have congenital cardiac disease, resulting in in-utero hydrops fetalis and neonatal death.

### CASE REPORT

The patient, a 27-year-old $G_2P_{1001}$ white female, had an unremarkable pregnancy until 28 weeks gestation when she was noted to have an elevated

Address correspondence/reprint requests to Dr. William J. Watson, Perinatology, 1201 S. Euclid Avenue, Ste. 204, Sioux Falls, SD 57105.

Received April 10, 1995
Accepted July 5, 1995

blood pressure of 140/90 and 1+ proteinuria. In her previous pregnancy, she had delivered at term without complications. There was no history of birth defects in her family. She was immune to rubella. Her initial blood pressure during the pregnancy had been 120/70. Over the ensuing week, her blood pressure increased, despite bed rest at home, to 160/100 and her proteinuria rose to 2+. She was admitted for evaluation, at which time an ultrasound showed a fetus with nonimmune hydrops. The fetus had ascites and pleural effusions. A maternal antibody screen was negative. Cordocentesis showed a fetal hemoglobin of 11 mg/dl, and a fetal karyotype obtained from the fetal blood was 46, XY. Amniotic fluid was obtained for viral cultures. The initial ultrasound suggesting a possible fetal cardiac anomaly was subsequently confirmed by echocardiography. Detailed views of the fetal heart indicated moderate hypoplasia of the left ventricle and mitral stenosis. A maternal 24-h urine collection showed 1.4 g of protein in 24 h. Over the next 3 days of inpatient observation, the mother developed severe preeclampsia with abnormal liver function levels. An induction of labor resulted in the vaginal delivery of a 1,685-g male infant with Apgars of 1, 2, and 5 at 1, 5, and 10 min, respectively. Immediately after birth, the infant was intubated and ventilated. Despite maximum ventilatory support with 100% oxygen, the infant had severe hypercapnia and persistent hypoxemia. The infant developed disseminated coagulopathy which became rapidly progressive. After the parents were advised of the deteriorating situation, the ventilatory support was withdrawn and the infant died.

A viral culture was not obtained from the newborn. An autopsy showed severe mitral stenosis with left atrial hypoplasia, a stenotic foramen ovale, a markedly enlarged ductus arteriosus, and enlarged pulmonary veins. Mild aortic stenosis and left ventricular hypoplasia were also noted. A microscopic examination of the heart showed focal disorganization of the nuclei with focal calcification. No viral studies were done on the myocardial tissue.

The amniotic fluid for the initial viral cultures was placed into 6 cell-culture tubes: 2 tubes of A-549 cells, 2 tubes of human foreskin fiberblasts (at <20 passages), and 1 tube each of rhesus monkey kidney and African green monkey kidney cells. All tubes were observed daily for cytopathic effects. On day 11, a cytopathic effect was observed in both the A-549 and African green monkey cell lines which was consistent with enterovirus. Material scraped from the tubes was processed for electron microscopy. Enterovirus/parvovirus-like particles, 27 nm in diameter, were documented. The cell culture isolate was further characterized by neutralization studies as Coxsackie B-1. The maternal antibodies to Coxsackie B-1 virus were not measured.

### DISCUSSION

Coxsackievirus infection during pregnancy may cause transplacental infection in the fetus unrelated to the clinical severity of the disease in the mother. The spectrum of neonatal disease ranges from no sequela whatsoever to fatal encephalomyocarditis.[7] Hydrops due to myocarditis in a fetus with presumed coxsackievirus infection in utero has been reported.[8] Hydrops fetalis has been known to be associated with maternal preeclampsia, as observed in our patient.

Brown and Evans,[9] in a prospective study, demonstrated an association between the serologic evidence of Coxsackie B virus infection during the first trimester of pregnancy and the subsequent birth of infants with congenital heart disease. However, the documentation of cardiovascular malformations in their study was poor. In a careful review of the cardiovascular effects of congenital infections, Rosenberg[6] concluded that coxsackievirus infection is a known cause of fetal myocarditis, but not a proven cause of fetal anomalies.

It is interesting to speculate as to when the infection may have occurred in our patient. Because she had no clinical evidence of infection, definitively determining when the in utero infection occurred was not possible. However, if the virus caused the congenital malformation of the fetal heart, the infection must have occurred quite early in the gestation. The virus was isolated from this patient by amniocentesis at 28 weeks gestation. Since so few cases of coxsackievirus infection in utero have been reported, there is little information on how long the virus can be cultured after a primary infection. Magnius et al.[10] pointed out that enteroviruses may be excreted for several weeks after an acute illness. The finding of focal calcification on microscopic evaluation of the fetal heart and the lack of an inflammatory response suggest that infection in this fetus was not recent.

In following Coxsackie B virus infections during the first trimester, Rosenberg[6] noted that the incidence of congenital cardiac disease increases from approximately 5–7/1,000 live births to 9/1,000 live births. However, such observations do not prove teratogenesis, since other factors, such as maternal fever or medications taken for viral illness, may increase the incidence of malformations.

Other investigators have demonstrated the predilection of coxsackievirus B infections for myocardial tissue. The association of coxsackievirus antigen with myocarditis has been demonstrated by immunofluorescent studies.[5] Whether this type of myocarditis can result in teratogenesis is unproven. It is reasonable to hypothesize, however, that myocarditis occurring in the fetus during the early first trimester may interfere with the complex embryological development occurring at this time.

Most studies on coxsackievirus infection during pregnancy have relied on the maternal serologic evidence of infection. The isolation of virus from the amniotic fluid presents a stronger case for transplacental infection. The association of group B coxsackievirus infection with fetal congenital cardiac disease in this case report, although not conclusive, suggests that transplacental coxsackievirus infection may result in fetal congenital cardiovascular malformations.

In the evaluation of a mother whose infant is noted to have congenital cardiac disease by fetal echocardiography performed during pregnancy, an amniocentesis for fetal karyotype is often obtained to search for aneuploidy as a cause of such malformations. In such a case, it is reasonable to consider sending an aliquot of the fluid obtained during amniocentesis for viral culture, since such an infection may occur in an asymptomatic mother.

### REFERENCES

1. Sever JL, Huebner RJ, Castellano GA, et al.: Serologic diagnosis "en masse" with multiple antigens. Am Rev Respir Dis 88(2):342–349, 1963.
2. Cherry JD, Soriano F, Jahn CL: Search for perinatal viral infection. Am J Dis Child 116:245–250, 1968.
3. Amstey MS, Miller RK, Menegus MA, di Sant'Agnese PA: Enterovirus in pregnant women and the perfused placenta. Am J Obstet Gynecol 158(4):775–782, 1988.
4. Modlin J: Perinatal echovirus infection: Insights from a literature review of 61 cases of serious infection and 16 outbreaks in nurseries. Rev Infect Dis 8:918–926, 1986.
5. Basso NGS, Fonseca MEF, Garsia AGP, Zuardi JAT, Silva MR, Outani H: Enterovirus isolation from foetal and placental tissues. Acta Virol 34:49–57, 1990.
6. Rosenberg HS: Cardiovascular effects of congenital infections. Am J Cardiovasc Pathol 1(2):147–156, 1987.
7. Modlin JF: Perinatal echovirus and group B coxsackievirus infections. Clin Perinatol 15(2):233–246, 1988.
8. Benirschke K, Swartz WH, Leopold G: Hydrops due to myocarditis in a fetus. Am J Cardiovasc Pathol 1:131–133, 1986.
9. Brown GC, Evans EN: Serologic evidence of coxsackievirus etiology of congenital heart disease. JAMA 199: 183–187, 1967.
10. Magnius L, Sterner G, Enocksson E: Infections with echoviruses and coxsackieviruses in late pregnancy. Scand J Infect Dis 71(Suppl):53–57, 1990.
